# Slow cryopreservation is not superior to vitrification in human spermatozoa; an experimental controlled study

**Published:** 2015-10

**Authors:** Mohamed Shehata Ali Mohamed

**Affiliations:** *Women Hospital, Faculty of Medicine, University of Cologne. Kerpener Straße 62, 50937 Cologne, Germany.*

**Keywords:** Human spermatozoa, Cryopreservation, Vitrification, Sperm viability, Mitochondrial membrane potential.

## Abstract

**Background:**

Spermatozoa cryopreservation is used for the management of infertility and some other medical conditions. The routinely applied cryopreservation technique depends on permeating cryoprotectants, whose toxic effects have raised the attention towards permeating cryoprotectants-free vitrification technique.

**Objective:**

To compare between the application of slow cryopreservation and vitrification on human spermatozoa.

**Materials and Methods:**

This was an experimental controlled study involving 33 human semen samples, where each sample was divided into three equal parts; fresh control, conventional slow freezing, and permeating cryoprotectants-free vitrification. Viability and mitochondrial membrane potential (MMP) of control and post-thawing spermatozoa were assessed with the sperm viability kit and the JC-1 kit, respectively, using fluorescence-activated cell sorting analysis.

**Results:**

Significant reduction of the progressive motility, viability and MMP was observed by the procedure of freezing and thawing, while there was not any significant difference between both cryopreservation techniques. Cryopreservation resulted in 48% reduction of the percentage of viable spermatozoa and 54.5% rise in the percentage of dead spermatozoa. In addition, high MMP was reduced by 24% and low MMP was increased by 34.75% in response to freezing and thawing. Progressive motility of spermatozoa was correlated significantly positive with high MMP and significantly negative with low MMP in control as well as post-thawing specimens (r=0.8881/ -0.8412, 0.7461/ -0.7510 and 0.7603/ -0.7839 for control, slow and vitrification respectively, p=0.0001).

**Conclusion:**

Although both cryopreservation techniques have similar results, vitrification is faster, easier and associated with less toxicity and costs. Thus, vitrification is recommended for the clinical application.

## Introduction

Conventional slow freezing of spermatozoa is commonly used for cryopreservation of both ejaculated and surgically retrieved spermatozoa for preservation of fertility before cancer treatment, in severe male factor infertility (obstructive azoospermia) and for establishment of donor banks. Cryopreservation of spermatozoa is therefore an important part of a successful assisted reproductive technology program (1).

Slow freezing of spermatozoa is commonly performed by stepwise manual or continuous programmed freezing of vials or straws, containing a mixture of cryoprotectants and spermatozoa, to subzero temperatures (1). To improve the outcome of spermatozoa cryopreservation, many attempts have been made, which relied on usage of various cryoprotective agents and manipulating the rate of cooling and warming (2). The permeating cryoprotectants, which are used for routine slow spermatozoa cryopreservation, are able to penetrate the cell membrane and protect against the intracellular ice formation. However, this could be associated with much toxicity and additional stress with the introduction and removal of these compounds before and after freezing and thawing (3). Traditionally used cryoprotective solutions are composed mainly of egg-yolk, which potentially increases the risk of transmission of diseases between species, and glycerol, which is considered a toxic compound (4). To avoid the negative influence of permeating cryoprotectants on human spermatozoa, the vitrification technique has recently been developed. This technique uses 0.25 M sucrose as a non-permeating cryoprotectant. Accordingly, the comparison between conventional slow freezing and vitrification, for the physiological parameters of post-thawing spermatozoa, has become an important target for investigations (5).

Many fluorescent probes have been used for assessment of spermatozoa physiological parameters. The fluorescence of these compounds can be estimated using fluorescence microscopy or flow cytometry. However, usage of flow cytometry is more recommended because it is an objective and more accurate assessment tool (6).

One of the important physiological parameters that reflect the quality of semen before and after cryopreservation is spermatozoa viability, which can be assessed with the use of many fluorescent probes. However, the SYBR-14/PI combination has been found to be more sensitive than other fluorescent probes (7).

Another important sperm parameter to be investigated is the mitochondrial membrane potential (MMP), which is considered a reliable indicator of spermatozoa mitochondrial functional status and cellular health (8). JC1, which is a lipophilic cationic dye, was found to be able to selectively enter into mitochondria and reversibly change colour according to changes in MMP (9).

Yoon *et al.* reported significant reduction of the physiological parameters of bull spermatozoa in response to cryopreservation, declaring that the freezing and thawing phases are the most critical steps (10). Jiménez-Rabadán *et al.* demonstrated that the vitrificantion of ram spermatozoa with dilutions of sucrose and glycerol had a drastic effect on the quality of the semen, which was partially improved by the addition of egg-yolk (11).

Although Moraes *et al*. have optimized the spermatozoa vitrification protocol to improve the practical outcome (12), Agha-Rahimi *et al*. reported the similarity between slow and rapid cryopreservation of normospermic human spermatozoa regarding the effects on sperm parameters, DNA fragmentation and hyaluronan binding (13).

The aim of the present study was to evaluate the described vitrification protocol, in comparison to the described slow cryopreservation protocol, regarding the effect on progressive motility, viability and mitochondrial functional status, using human spermatozoa and objective Fluorescence-activated cell sorting (FACS) analysis.

## Materials and methods

**Patients**

The study is an experimental controlled study, which was approved by the local institutional ethics committee (application Nr. 01-106, Ethics Committee, University of Cologne). After informing consents had been obtained, the semen samples were collected according to world health organization (WHO) criteria from 33 male subjects between 25 and 40 years of age. The samples were collected by masturbation after at least 48 hours of sexual abstinence. The donors were donating their sperms or patients attending the Infertility Clinic, University of Cologne. The samples were collected between April and October 2013.

Semen analysis was performed according to the published guidelines of the WHO (1) and the routinely applied techniques in our IVF laboratory. Samples were classified according to the following lower reference limits: 15 million spermatozoa/ml, 32% progressive motility and a minimum of 4% morphologically normal spermatozoa. Normozoospermic and oligoasthenozoospermic specimens were included in the study. Azoospermic and microbiologically contaminated semen specimens were excluded from the study. One part of the investigation was carried out on spermatozoa prepared by using the swim-up technique, as it allows for the selection of the most active, viable and morphologically normal fraction of ejaculated spermatozoa (14). While the other part was carried out on spermatozoa washed from seminal plasma to have a better understanding to which extent non-motile spermatozoa and non- spermatozoa cells (which are usually not included in swim-up preparation) could alter the viability or MMP percentages in samples before and after freezing and thawing. Freezing of whole semen was not considered in this study because the inclusion of the seminal plasma is known to be protective during cryopreservation while the target of the experiments was to assess the effects of the cryopreservation techniques on the processed semen, to get information regarding the degree of cellular affection.

**Figure 1:**
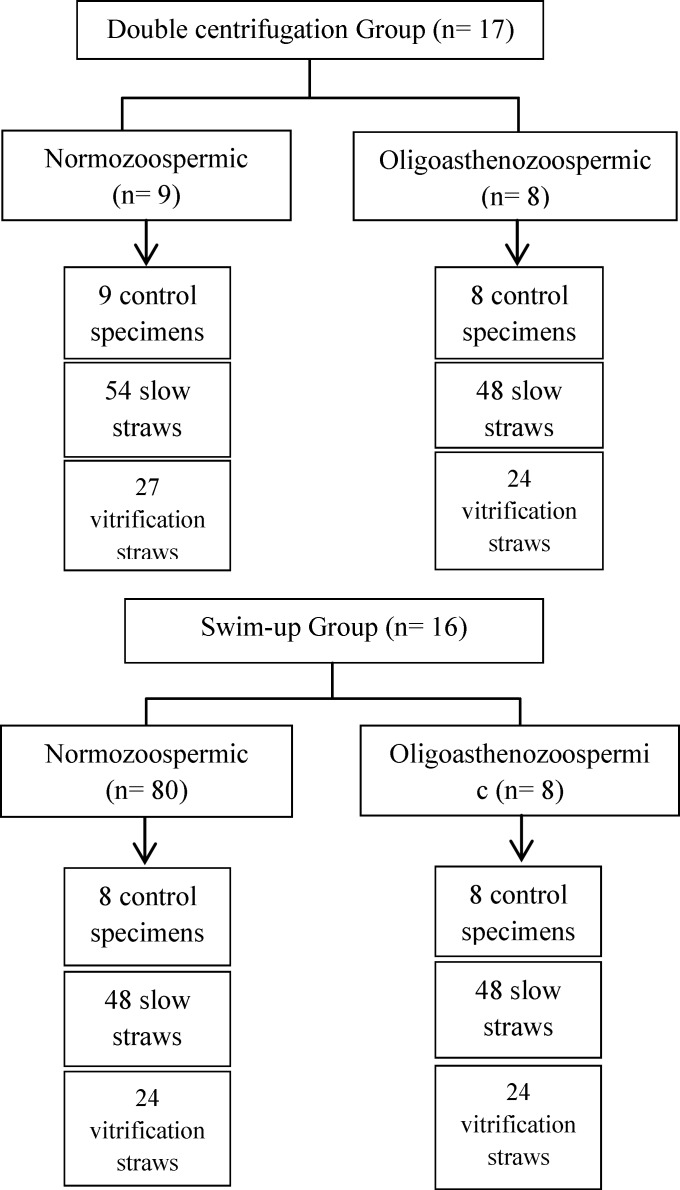
Diagrammatic representation of the study design

Each swim-up-prepared or double centrifuged sample of spermatozoa was divided into three equal parts:

1) fresh control, 2) conventional slow freezing and 3) vitrification (Figure 1).

This ensures careful randomization and guarantees that the obtained results would be optimal for comparing the effects of freezing and thawing regardless of other effects of preparation because it was equally applied for all specimens.

**Cryopreservation techniques**

**Double centrifugation spermatozoa preparation technique (DC)**

Semen sample was diluted 1:2 with pre-warmed (37^º ^C) Quinn’s sperm wash medium (Sage Media, Trumbull, CT, USA) and transferred into a conical centrifuge tube (Becton Dickinson, NJ, USA) and centrifuged at 300 g for 10 minutes. The supernatant was carefully removed and discarded. The sperm pellet was resuspended in 1 ml of the same medium by gentle pipetting, followed by centrifugation again for 10 minutes at 300 g. After removing and discarding the supernatant, the pellet was resuspended in variable volumes of Human Tubal Fluid medium (HTM) (Irvin Scientific, Barcelona, Spain) supplemented with 1% synthetic serum substitute (SSS, Irvine Scientific, Barcelona, Spain), by gentle pipetting, to achieve the desired spermatozoa end-concentration (15 x 10^6^ /ml).

**Swim-up spermatozoa preparation technique**

Semen sample was diluted 1: 2 with pre-warmed (37^º^C) Quinn’s sperm wash medium (Sage Media, Trumbull, CT, USA) and transferred into a conical centrifuge tube (Becton Dickinson, NJ, USA) and centrifuged at 300 g for 10 minutes. The supernatant was carefully removed and discarded. The sperm pellet was resuspended in 1 ml of the same medium by gentle pipetting, followed by centrifugation again for 10 minutes at 300 g. After removing and discarding the supernatant, 1 ml of pre-warmed (37^º^C) HTM +1% SSS was gently placed over the pellet, without disturbing it, followed by incubation for 60 minutes, at 37^º^C and 6% CO2 atmosphere, in oblique position (45^º^). After incubation, the tube was handled gently and returned to up-right position and the uppermost 500 µl medium was removed into a sterile Eppendorf tube where the highly motile sperms are present (1). Concentration and motility were re-examined under microscope.

**Slow freezing**

Spermatozoa were diluted 1: 2 in pre-warmed (37^º^C) commercial cryoprotective sperm freezing Medium (Irvin Scientific, Barcelona, Spain). The mixture was equilibrated for 10 minutes at room temperature and loaded in 0.25 ml standard insemination straws (Medical Technology GmbH). The straws were hermetically closed, then exposed horizontally to liquid nitrogen vapor (8 cm over the surface) for 30 minutes before being plunged into liquid nitrogen and stored for at least 24 hours. Although this manual freezing technique was effective, the cooling rate cannot be precisely determined. For warming, straws were taken out of liquid nitrogen and placed into 37^º^C water bath with gentle shaking till complete melting (7). Proper wash (dilution with pre-warmed 37^º^C Quinn’s sperm wash medium and centrifugation for 5 minutes at 300 g) was performed before subsequent processing to get rid of the cryoprotective medium (1, 6).

**“Straw in straw” vitrification technique**

For preparation of vitrification solution the 0.5 M stock of sucrose (MP Biomedicals, Illkirch, France) was dissolved into bi-distillate water (Berlin-Chemie, Berlin, Germany) followed by filtration through 0.22 µm filter (Millipore, Darmstadt, Germany). Aliquots were stored at -20^º^C till use. The vitrification solution was prepared ex-tempore as follows: 0.5 M sucrose was diluted 1:1 with HTM medium supplemented with 1% SSS, to achieve the 0.25 M sucrose end-concentration (5).

Prior to vitrification, spermatozoa were processed by swim-up or double centrifugation technique and, thereafter, the part meant for vitrification was centrifuged at 300 g for 10 minutes, followed by discarding of supernatant. The pellet was resuspended in vitrification medium to achieve the concentration of 15 x 10^6^ spermatozoa /ml with subsequent incubation for 5 minutes at 37^º^C and 5% CO2 (6).

The vitrification technique procedure involved a sterile ½ 0.25 ml insemination straw (MTG, Bruckberg, Germany) marked from one side which was filled with 100 µl of the sperm suspension and placed in a 0.5 ml plastic straw (MTG, Bruckberg, Germany) which was hermetically closed at both sides and directly plunged into liquid nitrogen. All manipulations were done strictly in horizontal position to avoid leakage of the sperm suspension between both straws. The straws were stored for at least 24 hours. Warming of vitrified probes was done as follows: the end of the 0.5 ml straw was cut in front of the marked ½ 0.25 ml insemination straw, while still placed in liquid nitrogen. The 0.25 ml straw was taken out and inserted into 15 ml plastic tube containing 5 ml HTM pre-warmed to 42^°^C, and supplemented with 1% SSS, followed by immediate gentle vortex and incubation at 37^°^C and 5% CO2 for 5 minutes. Afterwards, the 15 ml tubes were centrifuged at 300 g for 10 minutes. The supernatant was discarded and the pellet was resuspended in pre- warmed supplemented medium (37^º^C) to achieve the desired spermatozoa concentration.

**Sperm parameters assessment techniques**

**Evaluation of spermatozoa motility**

Motility of spermatozoa was assessed immediately after ejaculation and after each spermatozoa preparation technique (double-centrifugation and swim-up processing) and after thawing using the Makler’s counting chamber (0.01 mm^2^ and 10µm deep). Motility was estimated under the light microscope (Ziess, Goettingen, Germany) at 400 X magnification. Only spermatozoa with progressive motility, categories ‘a’ (rapid and regular forward progression) and ‘b’ (moderate, slow, or sluggish forward progression) according to the WHO (1), were assessed. The percentage of progressively (a + b) motile spermatozoa was determined according to the following equation; (a + b motile sperms/ total sperms) x 100.

**Flow cytometry**

For flow cytometric analysis, FACS Calibur (Becton Dickinson, NJ, USA) was set at various excitation/emission wavelengths according to kits’ instructions. 10000 events for each sample, including the blank control, were recorded. The data were analyzed using Cell Quest ^TM^ software (BD Biosciences, NJ, USA) using quadrants’ and regions’ states and the percentages of stained spermatozoa were determined.

**Sperm viability test**

The samples were processed using flow cytometry after SYBR-14 and Propidium Iodide (PI) staining using dead/live sperm viability kit (Molecular Probes Inc., OR, USA). Spermatozoa suspension was diluted to 1 x 10^6^ /ml and five microliters of SYBR-14 (1: 50 dilution) were added to 1 ml spermatozoa suspension (0.4 μM final concentration) followed by incubation for 10-15 minutes at 37^º^C. Five microliters of PI were added to the same suspension (0.5 μM final concentration) followed by incubation for 10-15 minutes at 37^º^C. Viable spermatozoa stained green with SYBR-14 were detected on FL1 channel while dead sperms stained red with PI were detected on FL2 channel, with compensation adjusted manually. When bound to DNA, the fluorescence emission maxima of SYBR-14 and PI are 516 nm and 617 nm, respectively. SYBR-14 is a membrane- permeant and non-fluorescent compound, which is immediately deacylated and converted into high fluorescent compounds by intracellular esterases. These green fluorochromes are maintained intracellular by intact membranes. As plasma membranes deteriorate at cell death, cells lose their ability to resist the influx of red fluorescent PI which replaces or quenches green fluorochromes.

**Sperm MMP**

The spermatozoa were stained with JC1 mitochondrial membrane potential sensor (Biotium, Hayward, CA, USA) according to kit instructions and processed using flow cytometry. One ml spermatozoa suspension of 1 x 10^6^ /ml concentration was centrifuged at 400 g for 10 minutes. The supernatant was discarded and the pellet was resuspended in 0.5 ml of "1XMIT-E" staining solution (prepared according to kit instructions, shortly; lyophilized material was dissolved into 125 µl of DMSO at room temperature) and incubated at 37^º^C for 15 minutes. Afterwards, centrifugation at 400 g for 10 minutes was done and the pellet was resuspended in 1ml of 1X assay buffer (provided in the kit without sufficient information about its composition) followed by centrifugation at 400 g for 10 minutes for washing. This washing process was repeated another two times before spermatozoa were re-suspended in 1 ml of HTM + 1% SSS and analyzed by FACS Calibur.

In non-damaged cells with high MMP (HMMP), JC1 spontaneously forms complexes known as J-aggregates with intense red fluorescence. Whereas in unhealthy cells with low MMP (LMMP), JC1 remains in the monomeric form, which shows only green fluorescence (peak emission at 527 nm) which is measured in the FL1 channel (530 nm).

JC1 aggregates show a red spectral shift (peak emission at 590 nm), and are measured in the FL2 channel (585 nm). Meanwhile, cells with altered mitochondrial function will remain bright in the FL1 channel, but will have reduced FL2 intensity.

**Statistical analysis**

For statistical analysis, Excel data sheet (Microsoft Office 2007) for calculation of mean and standard deviation (SD) was used. Comparison between different groups of results was done using the Prism6Demo program for determination of significant differences and correlations between motility and MMP as well as between viability and MMP. Non paired T- test was selected as the data showed a normal distribution graph. P-values less than 0.05 were considered significant.

## Results

**Sperm motility**

**Figure 2:**
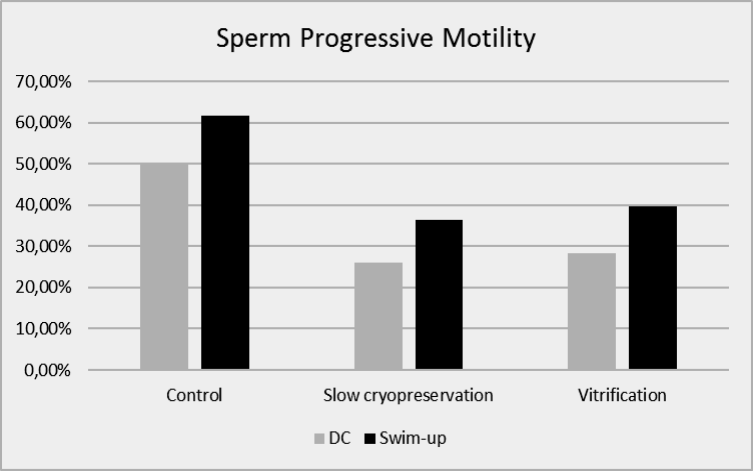
Comparison among averages of progressive motility of spermatozoa before and after cryopreservation. The rates of the treatment groups showed significant differences with the corresponding control (p< 0.05). No significant differences were found between the corresponding treatment groups (p> 0.05).

The process of freezing and thawing markedly affected the sperm motility as shown in figure 2. While the control non-frozen spermatozoa showed 56% progressive motility (50.39±10.24% in case of double centrifugation and 61.76±8.35% in case of swim-up), this percentage was reduced by about 45% to reach 31% in case of slow cryopreservation (26±3.98% in case of double centrifugation and 36.31±5.30% in case of swim- up), and by about 40% to reach 34% in case of vitrification (28.41±5.05% in case of double centrifugation and 39.69±5.61% in case of swim-up) (Figure 2). These reductions were highly significant (p<0.00). However, the differences between both cryopreservation techniques were non- significant (p=0.09 in case of swim-up and p=0.13 in case of DC).

**Sperm viability (cell membrane integrity)**

**Figure 3:**
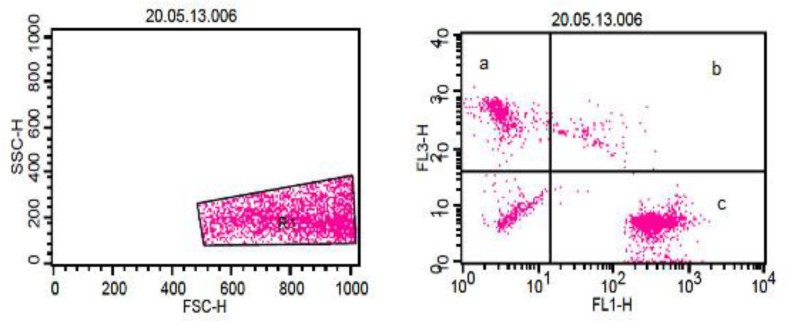
Detection of viable and dead spermatozoa with fluorescence-activated cell sorting analysis. R1 indicates selection of spermatozoa population. Viable spermatozoa stained green with SYBR-14 were detected on FL1 channel ‘c’. Dead spermatozoa stained red with PI were detected on FL2 channel ‘a’. Events in between are double stained spermatozoa ‘b’, and non-stained spermatozoa and debris

The influence of different cryopreservation techniques on spermatozoa viability was assessed using FACS analysis (Figure 3), which showed marked insult to cytoplasmic membranes and significant decrease in spermatozoa viability by the process of freezing and thawing.

**Double centrifugation group**

**Figure 4:**
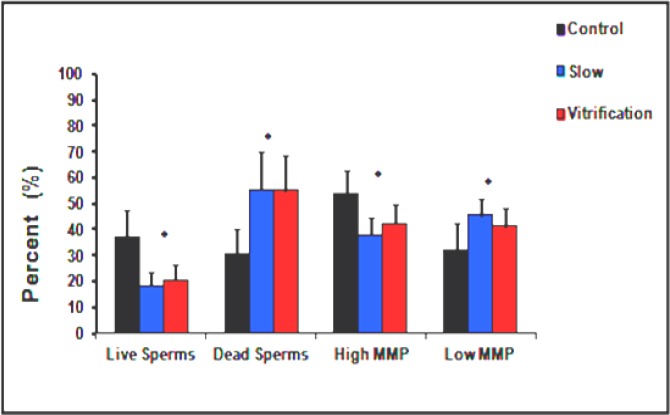
Influence of various cryopreservation techniques on the viability and mitochondrial membrane potential of spermatozoa prepared with double centrifugation. All rates in respective groups are significantly different (p< 0.05) except between columns marked with asterisks (p> 0.05).

Slow cryopreservation resulted in a 51% reduction in the average percentage of live spermatozoa (from 37.30±10.16 to 18.29±5.28). Meanwhile, vitrification resulted in a 45% reduction in the average percent of live spermatozoa (from 37.30±10.16 to 20.47±5.87). In addition, both cryopreservation techniques resulted in about 46% (from 30.62±9.39 to 55.35±14.87 and 55.00±13.50) rise in the average percentage of dead spermatozoa (Figure 4).

**Swim-up group**

**Figure 5:**
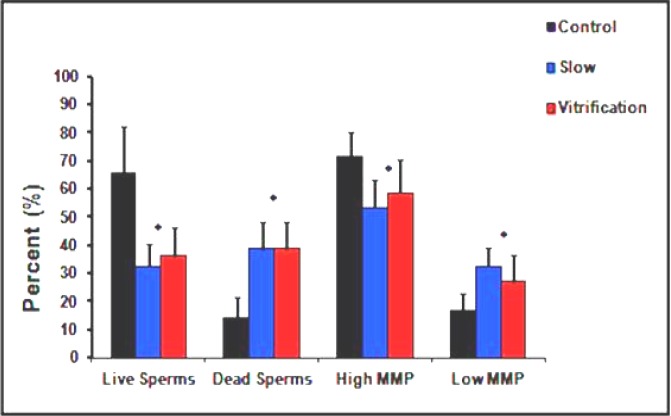
Influence of various cryopreservation techniques on the viability and mitochondrial membrane potential of spermatozoa prepared with swim-up. All rates in respective groups are significantly different (p< 0.05) except between columns marked with asterisks (p> 0.05).

Slow cryopreservation resulted in a 51% reduction in the average percentage of live spermatozoa (from 65.36±16.45 to 32.31±7.91). Meanwhile, vitrification resulted in a 45% reduction in the average percentage of live spermatozoa (from 65.36±16.45 to 35.88±10.63). In addition, both cryopreservation techniques resulted in 63% (from 14.28±7.52 to 38.44±9.76 and 38.44±9.82) rise in the average percentage of dead spermatozoa (Figure 5).

In both groups, the rates of change in percentages of live and dead spermatozoa, showed significant differences in comparison to control non- frozen spermatozoa (p<0.001). However, all rates showed insignificant differences between both cryopreservation techniques (Plive = 0.29 in case of swim-up and 0.26 in case of DC) (Pdead = 1.00 in case of swim-up and 0.94 in case of DC).

**Sperm MMP**

**Figure 6:**
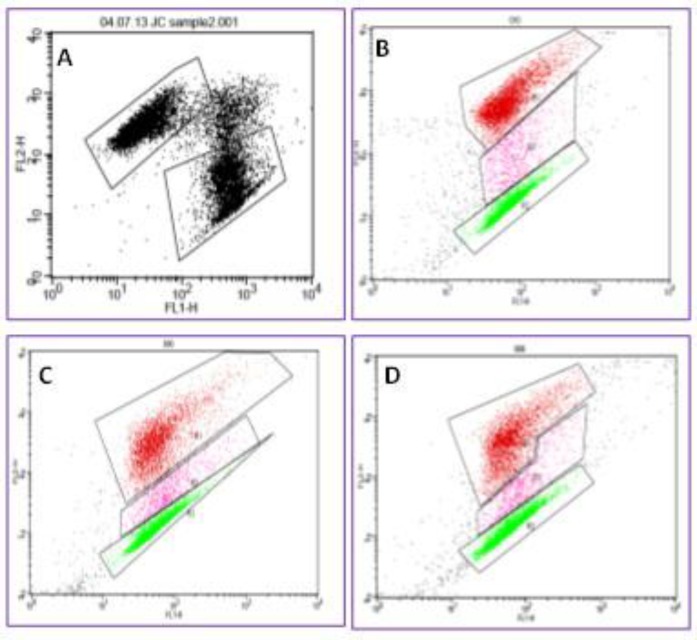
Detection of spermatozoa high and low mitochondrial membrane potential with fluorescence-activated cell sorting analysis. In non-damaged cells with high mitochondrial membrane potential, JC1 spontaneously forms complexes known as J-aggregates with intense red fluorescence. Whereas in unhealthy cells with low mitochondrial membrane potential, JC1 remains in the monomeric form, which exhibits green fluorescence (peak emission at 527 nm), which is measured in the FL1 channel (530 nm). JC1 aggregates show a red spectral shift (peak emission at 590 nm), and are measured in the FL2 channel (585 nm).

The influence of both cryopreservation techniques on spermatozoa MMP was assessed by FACS analysis (Figure 6), which showed significant post-thawing changes.

**Double centrifugation group**

Slow cryopreservation resulted in 30% (from 53.53±9.48 to 37.94±6.68) reduction in the average percent of HMMP, and 30% (from 31.82±10.77 to 45.65±6.63) rise in the average percent of LMMP. Meanwhile, vitrification resulted in 21% (from 53.53±9.48 to 42.35±7.27) reduction in the average percent of HMMP and 24% (from 31.82±10.77 to 41.41±7.06) rise in the average percent of LMMP (Figure 4).

**Swim-up group**

Slow cryopreservation resulted in 26% (from 71.63±8.66 to 53.25±9.77) reduction in the average percent of HMMP, and 48% (from 16.88±5.86 to 32.44±6.54) rise in the average percentage of LMMP. Meanwhile, vitrification resulted in 19% (from 71.63±8.66 to 58.44±11.93) reduction in the average percentage of HMMP and 37% (from 16.88±5.86 to 26.81±9.49) rise in the average percent of LMMP (Figure 5).

In both groups the changes in spermatozoa MMP showed significant differences in comparison to control non-frozen spermatozoa (p<0.001). However, all rates showed insignificant differences between both cryopreservation techniques (PHMMP=0.18 in case of swim-up and 0.07 in case of DC) (PLMMP=0.06 in case of swim-up and 0.08 in case of DC).

**Correlation among sperm motility, mitochondrial membrane potential and viability**

Progressive motility of spermatozoa was found to correlate significantly positive with HMMP and significantly negative with LMMP, in control as well as post-thawing specimens (r = 0.8881/-0.8412, 0.7461/-0.7510 and 0.7603/-0.7839 for control, slow and vitrification respectively, p= 0.0001). Also, spermatozoa viability showed significant positive correlation with HMMP and significant negative correlation with LMMP, in control as well as post-thawing specimens (p= 0.0001).

**Comparison between influence of the ejaculate treatment before cryo-preservation on spermatozoa viability and mitochondrial membrane potential after cryopreservation**

**Figure 7:**
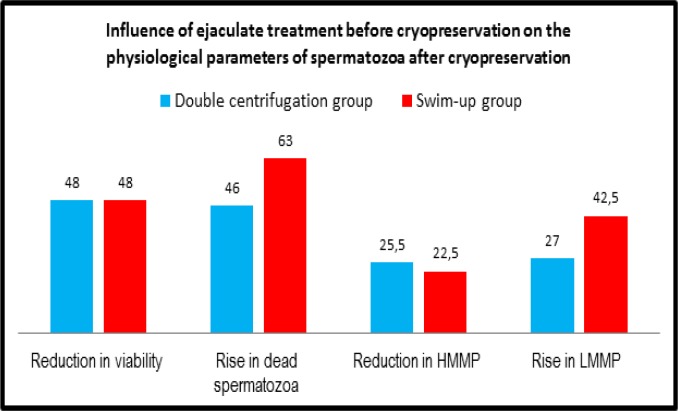
Influence of the ejaculate treatment before cryopreservation on the physiological parameters of spermatozoa after cryopreservation

As shown in figure 7, the rates of reduction in spermatozoa viability by both cryopreservation techniques were similar for both preparation techniques (double centrifugation and swim-up). However, the rate of rise in dead spermatozoa was obviously higher in case of swim- up than in case of double centrifugation. Meanwhile, the rate of reduction in HMMP was slightly higher in case of double centrifugation than in case of swim-up. In addition, the rate of rise in LMMP was obviously higher in case of swim-up than in case of double centrifugation (Figure 7).

## Discussion

Spermatozoa cryopreservation is routinely achieved using the conventional slow freezing technique. However, permeating cryoprotectants-free vitrification has recently been introduced (12, 15). To compare between both techniques, much care is given to sperm motility because this variable is the first to be affected (16). Sperm motility is essential for migration through the female reproductive tract (internal fertilization) or migration in water (external fertilization) to reach the ovum (17). Motility is also important for sperms in order to penetrate the extracellular matrix surrounding ova. It is well stated that cryopreservation results in significant impairment of sperm motility, though the mechanism remains unclear. However, mechanical, physical and or chemical etiologies have been suggested (16, 18).

Intracellular and extracellular formation of ice crystals and osmotic damage due to extensive cell shrinkage are major hazards of conventional cryopreservation. Further deterioration of the cellular viability can occur during subsequent re-warming and thawing, due to osmotic swelling, which affects the post-thawing motility (18, 19).

Conventional slow cryopreservation causes extensive chemical and physical damage to sperm cell membrane due to changes in lipid-phase transition, increased lipid peroxidation and production of reactive oxygen species (ROS) (20). All these events could result in diminished sperm motility (21). It was suggested that diminished spermatozoa motility occurs during cooling due to increased production of ROS and lack of the antioxidant defence activity, as well as due to the structural damage of the sperm cytoskeletal elements. However, further reduction of motility occurs during thawing (22).

As the cytoskeletal elements are sensitive to temperature changes, the presence of extracellular ice during cellular freezing results in depolymerization of the fine cortical actin cytoskeleton mesh-work and fragmentation of thicker bundles (23). In the present study, progressive motility of spermatozoa was significantly reduced after cryopreservation. In fresh control specimens, the percentage of progressive motility showed significant positive correlation with HMMP and significant negative correlation with LMMP. Those significant correlations were kept after freezing and thawing by both techniques, denoting that MMP (which reflects the functional state of spermatozoa mitochondria and ATP generation) might directly affect motility of spermatozoa before and after freezing and thawing.

Although the percentage of progressive motility of vitrified spermatozoa was found to be slightly higher than that of conventional slow freezing, there was no significant difference between both techniques in this regards. In general, cellular motility depends mainly on intact functional cytoskeleton system and energy supplementation in the form of ATP (24). Accordingly, the observed reduction in spermatozoa motility might be explained by the affection of cytoskeleton elements during the process of freezing and thawing, in addition to the reduction in ATP production. These results go with the results shown before by Isachenko *et al* (8-11, 24, 25) and Kim *et al* (26), suggesting extra- hazardous effects of permeating cryoprotectants used in conventional slow cryopreservation. In addition, some improvement of motility was observed with ATP supplementation before slow freezing, but without corresponding improvement of MMP (26).

The slow cryopreservation protocol has some potentially damaging stresses: firstly; the change in temperature, secondly; the osmotic and toxic stresses of permeating cryoprotectants, and thirdly; the formation and dissolution of ice in the extracellular environment. It was observed that spermatozoa cryopreservation induces a lethal cellular stress proportional to the rate of cooling (27). Mainly, the changes of membrane lipids, which occur during freezing, are responsible for the lethal stresses of cryopreservation (28).

Permeating cryoprotectants are used to prevent excessive cell shrinkage and intracellular ice formation during freezing. However, their effectiveness can only be achieved with low cooling rate, which might be harmful by itself (20). Increasing the cooling rate to decrease the lethal membrane lipids’ changes, would decrease the protective effects of permeating cryoprotectants. Based on that, permeating cryoprotectants-free vitrification would be expected to result in better spermatozoa viability. Thus, the exclusion of permeating cryoprotectants, the modification of the cooling rate and the addition of carbohydrates and proteins to the freezing medium, prevented the formation of big extracellular ice crystals and stabilized sperm cell membranes (24, 25). However, according to literature, and whatever the technique used, even if the process of spermatozoa cryopreservation was optimized and the cell death was minimized, there will still be a proportion of cells which fail to survive. Therefore, the concentration should be put on the functional abilities of the surviving population (27).

The routinely performed semen analysis is mainly dependent on microscopic and manual evaluation techniques, which are highly affected by subjective variations. However, the objective techniques, such as FACS analyses, are currently introduced and recommended, which involve the use of many fluorescent probes (29). The fluorescence of these compounds can be estimated using fluorescence microscopy or flow cytometry. Flow cytometry enables objective and accurate analysis, where a big number of spermatozoa (10000) can be analysed in small volumes of samples, in a short time. This is considerably more than the total of 200 cells generally observed by microscopic analysis (30).

The integrity of sperm membranes is a necessary condition for maintaining spermatozoa viability and for penetration of the oocytes (31). When semen is frozen, cells are exposed to a cold shock, formation of ice crystals, and cellular dehydration, which all cause irreversible damage (32). The results of the present study show significant reduction in viability and cell membrane integrity of spermatozoa after freezing and thawing. This was similarly observed in previous research work for slow conventional freezing (26, 31) as well as for cryoprotectants-free vitrification (8-13, 24, 25).

Although the percentages of live spermatozoa after vitrification were found to be slightly higher than those after conventional slow freezing, the percentages of dead spermatozoa after both cryopreservation techniques were similar, and there were no significant differences between both techniques. These results show that the presence of permeating cryoprotectants in the cryoprotective solution did not influence the viability of spermatozoa after application of both cryopreservation techniques.

In all living cells, oxygen metabolism results in production of reactive oxygen species (ROS) which are very harmful to DNA, membrane lipids and other cellular organelles. However, under physiological conditions, ROS are kept in balance with antioxidants. When this fine balance is disturbed, a state of oxidative stress results, which may lead to a serious or lethal outcome (33). In spermatozoa, the short-lived ROS are involved in control of some physiological functions including capacitation, acrosome reaction and fertilization (34).

ROS production was found to negatively correlate with MMP in normozoospermic, asthenozoospermic and oligoastheno-zoospermic samples, and the reduction of MMP by the process of cryopreservation is thought to result from increased ROS production (35). In the present study, the percentage of HMMP was significantly reduced, while the percentage of LMMP was significantly increased, by both cryopreservation techniques. Similar findings showing a significant reduction of MMP and sperm viability/membrane integrity after freezing and thawing were reported before, with an observed increase of the retrieved sperm motility as the cooling rate was increased (36). However, the direct comparison between both cryopreservation techniques showed no significant differences, though the results were slightly better for vitrification.

The spermatozoa preparation technique can also influence the physiological parameters before and after cryopreservation (37, 38). In the present study two types of spermatozoa preparation techniques were applied; double centrifugation and conventional swim-up. The pattern of differences between both groups after cryopreservation showed no difference in survived cells, however, the percentage of dead cells were higher in swim-up.

Taking into account that double centrifugation technique eliminates seminal plasma while some non-sperm cells might not be eliminated, the increased rise in dead cells by cryopreservation in case of swim-up may denote more sensitivity of spermatozoa, and less sensitivity of the remaining non sperm cells, to the process of cryopreservation. This might also explain the observed pattern of change in MMP. While the reduction rates of HMMP by cryopreservation were close for both preparation techniques (though slightly less in case of swim-up), the rate of increase of LMMP was obliviously higher in case of swim-up.

In the present study, the comment on the effects of spermatozoa preparation techniques on the outcome of cryopreservation could be only descriptive (Figure 7). That is because the various preparation techniques were applied on different samples (17 ejaculates were prepared with double centrifugation technique versus 16 ejaculates were prepared with swim-up technique). Nevertheless, the results of the present study go with the concept that, the inclusion of seminal plasma and non-sperm cells of the ejaculate might decrease the sensitivity of spermatozoa to the cryopreservation procedure. However, for precise investigations in this regards, a bigger study with more samples should be conducted, where the same probe would be divided into two equal parts and every part would be subjected to the preparation technique of interest followed by cryopreservation.

Recently, FACS analysis has replaced other subjective methods for routine semen analysis (18). The experience obtained through the present study showed better accuracy of FACS analysis, especially when applied for swim-up prepared spermatozoa, because the DNA and mitochondria containing particles in this case would be mainly spermatozoa. The value of the objective analysis over the subjective analysis can be clearly observed through the results of the present study as the percentages of progressive motility detected by the subjective counting were higher than the percentages of living spermatozoa detected by objective FACS analysis. Although this appears non- sense, there are some explanations beyond those results; firstly, classifying and counting motile spermatozoa under microscope need much experience to be accurate and still would never be as accurate as objective automated tools. Secondly, the percentage of living spermatozoa was obtained by staining, out of 10000 events per dot plot, while the percentage of progressively motile spermatozoa was obtained by counting out of a maximum of 200 cells per field. Thirdly, counting under microscope involved only spermatozoa because they could be visually identified, however, the counted events by FACS in the present study might involve double stained spermatozoa, unstained spermatozoa, and/or cellular debris that fail to be eliminated during sample processing. In addition, other causes such as sensitiveness of staining and relative time lagging might be accused. However, all specimens before and after cryopreservation, were subjected to similar handling and assessed under similar conditions, which renders comparison between control and post-thawing spermatozoa scientifically reliable. In other words, the results of the subjective assessment of motility and the results of the objective FACS analysis of the present study should be considered independently though correlations between both assessments were valuable.

According to a common point of view the non-permeating cryoprotectants play the supporting role of permeating cryoprotectants. They bind extracellular water and at the same time exclude the harmful effects of permeating cryoprotectants (39). In general, the inclusion of osmotically active, non-permeating compounds into the cryopreservation solution would lead to additional dehydration of the cells and, as a result, would decrease the toxic effects of the permeating cryoprotectants on intracellular structures. In addition, sugars can provide some stabilization to cell membranes (40). Nevertheless, vitrification allows avoiding the additional stresses caused by the addition and the removal of the permeating cryoprotectants, including the negative effects on the cells’ genetic material. Moreover, vitrification is accompanied with less cost (15). 

## Conclusion

The present study confirms the significant affection of the progressive motility, viability and mitochondrial membrane potential of human spermatozoa by cryopreservation, however, without meaningful differences between the conventional slow and the vitrification cryopreservation techniques. As vitrification has the tendency to avoid the toxic hazards of the permeating cryoprotectants, is easier to perform, is associated with less cost and is less time consuming, the application of vitrification in the practice of assisted reproductive technology and cryopreservation of spermatozoa is expected to improve the clinical outcome. 

## Note

***This study is extracted from MD thesis (Mohamed Shehata Ali Mohamed).***
